# Arkadia Enhances Nodal/TGF-β Signaling by Coupling Phospho-Smad2/3 Activity and Turnover

**DOI:** 10.1371/journal.pbio.0050067

**Published:** 2007-03-06

**Authors:** Konstantinos J Mavrakis, Rebecca L Andrew, Kian Leong Lee, Chariklia Petropoulou, James E Dixon, Naveenan Navaratnam, Dominic P Norris, Vasso Episkopou

**Affiliations:** 1 Mammalian Neurogenesis, Medical Research Council, Clinical Sciences Centre, Imperial College School of Medicine, Hammersmith Hospital, London, United Kingdom; 2 Mammalian Genetics Unit, Medical Research Council, Harwell, United Kingdom; Osaka University, Japan

## Abstract

Regulation of transforming growth factor-β (TGF-β) signaling is critical in vertebrate development, as several members of the TGF-β family have been shown to act as morphogens, controlling a variety of cell fate decisions depending on concentration. Little is known about the role of intracellular regulation of the TGF-β pathway in development. E3 ubiquitin ligases target specific protein substrates for proteasome-mediated degradation, and several are implicated in signaling. We have shown that Arkadia, a nuclear RING-domain E3 ubiquitin ligase, is essential for a subset of Nodal functions in the embryo, but the molecular mechanism of its action in embryonic cells had not been addressed. Here, we find that Arkadia facilitates Nodal signaling broadly in the embryo, and that it is indispensable for cell fates that depend on maximum signaling. Loss of Arkadia in embryonic cells causes nuclear accumulation of phospho-Smad2/3 (P-Smad2/3), the effectors of Nodal signaling; however, these must be repressed or hypoactive as the expression of their direct target genes is reduced or lost. Molecular and functional analysis shows that Arkadia interacts with and ubiquitinates P-Smad2/3 causing their degradation, and that this is via the same domains required for enhancing their activity. Consistent with this dual function, introduction of Arkadia in homozygous null (−/−) embryonic stem cells activates the accumulated and hypoactive P-Smad2/3 at the expense of their abundance. *Arkadia*−/− cells, like *Smad2−/−* cells, cannot form foregut and prechordal plate in chimeras, confirming this functional interaction in vivo. As Arkadia overexpression never represses, and in some cells enhances signaling, the degradation of P-Smad2/3 by Arkadia cannot occur prior to their activation in the nucleus. Therefore, Arkadia provides a mechanism for signaling termination at the end of the cascade by coupling degradation of P-Smad2/3 with the activation of target gene transcription. This mechanism can account for achieving efficient and maximum Nodal signaling during embryogenesis and for rapid resetting of target gene promoters allowing cells to respond to dynamic changes in extracellular signals.

## Author Summary

In development, cells respond to secreted signals (called morphogens) by turning on or off sets of target genes. How does gene activity adjust quickly in response to rapidly changing extracellular signals? This should require efficient removal of old/used signaling effectors (signal-activated transcription factors) from the promoters of target genes to allow new ones to assume control. We previously discovered Arkadia, an E3 ubiquitin ligase, and showed that it is an essential factor for normal development. (Ubiquitin ligases trigger the addition of ubiquitin residues to proteins, typically marking them for degradation.) Here, we show that Arkadia is required for high activity of the major signaling pathway, TGF-β/Nodal. Arkadia has a dual role to degrade Smads, the TGF-β signaling effectors, and enhance their transcriptional activity. This coupling of degradation with activation provides a mechanism to ensure that only effectors “in use” are degraded, allowing the new ones to proceed. It is possible that very similar mechanisms operate in other pathways to establish dynamic regulation and efficient signaling, while their failure may be associated with developmental abnormalities and disease, including cancer.

## Abstract 3

Arkadia enhances TGF-β family activity by degrading its inhibitory Smads but also stimulating transcription of phospho-Smads.

## Introduction

Transforming growth factor-β (TGF-β) signaling controls a diverse set of cellular processes, including cell proliferation, differentiation, apoptosis, and specification of fate in vertebrate and invertebrate species. Disruption of signaling leads to developmental abnormalities and disease, including cancer. Activin and Nodal TGF-β ligands have been shown to act as morphogens in vertebrate development [1–4]. For example, in the mouse, Nodal is required for gastrulation, including development of the anterior primitive streak and the formation of the germ layers, endoderm and mesoderm [5,6]; for maintenance of pluripotency in the epiblast [7,8]; and for the specification of the anterior-posterior [9,10] and left-right axes [11]. Loss-of-function mutations in the *Nodal* gene, including enhancer deletions, lead to a reduction of *Nodal* RNA [12] and reveal that the highest level of Nodal signaling is required during gastrulation for the induction of the anterior primitive streak. This contains the precursors of the mammalian equivalent of the amphibian Spemann's organizer, and it gives rise to the anterior endoderm, the node, and the mesendoderm (notochord and prechordal plate), all of which are required for subsequent patterning of the vertebrate embryo [6]. Complementary experiments in *Xenopus* embryos, where increasing amounts of *Nodal* RNA are injected, show that it functions as a dose-dependent inducer and that the highest level induces Spemann's organizer [13]. The dynamic changes in the concentration of ligands, to elicit different cellular responses, demand that the responding cells have rapid turnover of the signaling-effectors and frequent refreshing of target gene promoters. Therefore, how TGF-β is regulated, and particularly, how signaling is terminated in the nucleus after gene transcription, is key in understanding cell fate decisions and patterning in vertebrate development.

TGF-β signals bind to cognate serine/threonine kinase receptors leading to phosphorylation and activation of the Smad family of signal transducers. Two different Smad signaling branches have been described. Ligands, like Activin, Nodal, Gdf1, Vg1, and TGFβ1 are transduced by the receptor-activated Smad2 and Smad3 (Smad2/3) [14,15]. The phosphorylated form of Smads (phospho-Smads [P-Smads]) complex with Smad4 and together translocate into the nucleus, where they function as transcription factors in association with DNA-binding partners such as FoxH1, Mixer, Jun/Fos, Runx, ATF3, and E2F4/5, etc., which provide target gene specificity [15]. In the mouse, loss-of-function mutations affecting core components of the Nodal signal transduction pathway give patterning and cell fate defects similar to that of *Nodal* itself [16–20].

Extracellular cofactors [21], antagonists [22,23], and proteases [24,25] have been shown to regulate Nodal activity during mouse development. However, little is known about the role of intracellular regulation in cells receiving Nodal. Intracellular regulators of the pathway include negative regulators such as inhibitory Smads (Smad6/7) that block TGF-β signaling by competing with Smads for association with the receptors or by targeting receptors for ubiquitin-mediated degradation [26–28]; in the nucleus, Ski and SnoN incorporate in the Smad DNA-binding complex to prevent them from binding to the transcriptional coactivator p300/CBP and repress transcription by recruiting histone deacetylase [29,30]. More recently, a phosphatase, PPM1A/PP2Ca, has been identified and is shown to de-phosphorylate P-Smad2/3 [31] and abrogate their signaling activity. Furthermore, proteasome-mediated degradation of ubiquitin-modified core components of the TGF-β signaling cascade has been shown to play a major role in controlling signaling output [32]. Poly-ubiquitination and proteasome-dependent degradation of proteins is one of the most prominent turnover mechanisms in the cell. Ubiquitination of protein substrates involves a cascade of enzymatic reactions. E3 ubiquitin ligases are the critical components responsible for the recognition of specific substrates for ubiquitination [33]. They are generally classified into the HECT- and RING-domain classes and exhibit substrate specificity [34,35]. Several ubiquitin ligases are known to reduce signaling by mediating the degradation of individual components of the pathway [28,36]. However, ligases that terminate signaling by degrading activated Smads (P-Smads) have not been identified.

One of the important unanswered questions is how long the activated Smads transcribe target genes and how the promoters are refreshed to allow rapid intracellular responses to dynamic changes in concentration of ligands. Both de-phosphorylation of P-Smads followed by cytoplasmic recycling [37] and proteasome-mediated degradation of P-Smads have been proposed to terminate signaling in the nucleus [38]. However, there was no explanation for how “used” versus “unused” activated effectors could be distinguished by these mechanisms. An obvious mechanism to limit the time that an effector works is to link its turnover with its ability to drive transcription.

We have shown previously that Arkadia, a nuclear RING-domain ubiquitin ligase, enhances Nodal signaling and is essential for the induction of the organizer/node [39,40]. In somatic tumor cell lines, Arkadia has been shown to enhance TGF-β signaling by ubiquitin-mediated degradation of Smad6/7 [41,42]. However, we show here that Arkadia functions by a different mechanism in embryonic cells. Specifically, we find that Arkadia directly ubiquitinates and degrades P-Smad2/3 and that this is coupled with their high activity. The link between activity and degradation provides a mechanism to ensure that only “used” P-Smad2/3 effectors are degraded and that signaling is terminated at the end of the cascade and not before. Therefore, Arkadia can account for rapid resetting of actively transcribed promoters, forcing transcription to rely only on fresh P-Smads and allowing cells to respond to dynamic changes in signaling. Similar mechanisms may operate in other signaling pathways in development to achieve peak efficiency and dynamic responses of cells during development.

## Results

### Arkadia Enhances Signaling in All Major Nodal-Dependent Developmental Events

**Figure 1:**
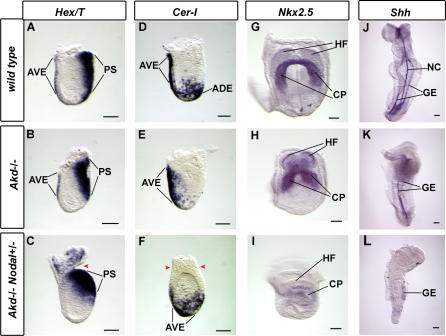
Arkadia Facilitates All Major Nodal-Dependent Developmental Events (A–F) In situ hybridization on 6.5 dpc embryos (mid- to late-streak stage) shown as lateral views with anterior to the left and posterior to the right, with probes (A–C), *Hex* and *Brachyury;* (D–F), *Cer-l.* In (C), *Akd−/− Nodal+/−* embryo showing lack of elongation and proximal positioning of the *Brachyury*–expressing primitive streak, as well as loss of *Hex* expression indicating absence of AVE. (E) An *Akd*−/− embryo with normal *Cer-l* expression in the AVE but no *Cer-l* expressing ADE. (F) An *Akd−/−*, *Nodal+/−* embryo showing a distal *Cer-l*-expressing domain indicating lack of AVE migration (three other embryos do not express *Cer-l,* not shown). Red arrowheads point to a constriction between extraembryonic and embryonic regions indicating incomplete anterior-posterior axis specification. (G–I) *Nkx2.5* probe on four-somite stage, 8.5 dpc embryo showing expression in the cardiac precursor tissue in the wild-type (G) and *Akd*−/− (H), but not in the *Akd−/−*, *Nodal+/−* embryo (I). (J–L) *Shh* probe on 12-somite stage embryos 9.5 dpc viewed from the ventral side with anterior toward the top. In the *Akd−/− Nodal+/−*embryo (L), note the reduction of *Shh* expression, the increased severity of the anterior truncation, and the absence of a morphologically distinguishable heart tube. PS, primitive streak; T, *Brachyury;* CP, cardiac precursors; HF, head folds; NC, notochord; GE, gut endoderm. Bars, 0.1 mm.

The phenotype of *Arkadia−/−* embryos consists of loss of anterior primitive streak derivatives (node, notochord, prechordal plate, and anterior definitive endoderm [ADE]/foregut) leading to anterior patterning defects including head truncations [39,40]. We have shown previously that while *Arkadia* or *Nodal* heterozygous (+/−) mice are normal, a small number of double heterozygotes for *Arkadia* and *Nodal* recapitulate the *Arkadia−/−* phenotype [39,40]. This suggested a functional interaction between Arkadia and Nodal. To investigate the extent of Arkadia's role in Nodal signaling, and in additional Nodal-dependent developmental events, we generated *Arkadia−/−* embryos with only one wild-type copy of the *Nodal* gene (*Akd−/−*, *Nodal+/−*). The majority of *Akd−/−*, *Nodal+/−* embryos that were analyzed (*n* = 19/33) exhibited phenotypes never observed in *Arkadia−/−* embryos. Using whole mount in situ hybridization, we performed marker analysis to define whether these phenotypes are Nodal-dependent (Figure 1).

Before gastrulation, Nodal signaling is responsible for anterior-posterior axis specification via the induction of the anterior visceral endoderm (AVE) domain at the distal tip of the mouse embryo and its migration to the prospective anterior [2,43]. Expression analysis of the AVE markers *Hex* [44] and *Cerl* [45] demonstrates that the *Arkadia−/−* embryos are always able to induce an AVE, which correctly migrates to the prospective anterior of the embryo (compare Figure 1A and 1D to 1B and 1E), while the primitive streak marker *Brachyury* is normally expressed posteriorly (Figure 1A and 1B). However, *Akd−/−*, *Nodal+/−* embryos have incomplete AVE-specific gene expression, as they are rarely able to induce an AVE (one out of seven) that expresses *Hex* (none out of two; Figure 1C), *Cerl* (one out of three; Figure 1F), or *Lefty1* (none out of two; unpublished data). Furthermore, in the *Akd−/−*, *Nodal+/−* embryos, the *Cerl*-expressing embryo AVE domain remains distal (Figure 1F) and the *Brachyury* domain remains proximal (Figure 1C), indicating that when the AVE is induced it cannot migrate to the anterior. In addition, more than 50% of the *Akd−/−*, *Nodal+/−* embryos examined at mid-streak stage have a constriction between the embryonic and extraembryonic compartments. This phenotype, which is not observed in *Arkadia−/−* embryos, is thought to be due to a failure of the AVE to migrate and define the anterior-posterior axis, leading to failure of primitive streak elongation and mesoderm formation along the embryonic-extraembryonic boundary [46]. These new phenotypes in *Arkadia−/−* embryos carrying only one *Nodal* wild-type allele indicates that Arkadia is also involved in AVE formation and suggests that it enhances Nodal signaling at pre-gastrulation stages.

During gastrulation, Nodal is responsible for the formation and patterning of endoderm and mesoderm. Cardiac mesoderm is considered an anterior mesodermal tissue [47], and *Arkadia−/−* embryos form heart [39]. In contrast, all *Akd−/−*, *Nodal+/−* embryos fail to form morphologically visible heart (*n* = 4; Figure 1L), as it is also shown by loss of *Nkx2.5* expression; one of the earliest markers of myocardial differentiation [48] (Figure 1G−1I), *Shh* expression (Figure 1J) marks the mesendoderm, the midline of the neural tube (floor plate), and the gut endoderm [49]. In *Arkadia−/−* embryos, *Shh* expression is reduced [39] due to the absence of mesendoderm and foregut, but it is present in the midgut and hindgut (Figure 1K). *Akd−/−*, *Nodal+/−* embryos, however, have a severe reduction in *Shh* expression indicating not only loss of mesendoderm but also of all endoderm (*n* = 2; Figure 1L), as confirmed by histological analysis (unpublished data). These new phenotypes seen in *Arkadia−/−* embryos carrying only one *Nodal* wild-type allele indicate that during gastrulation, Arkadia is involved in the formation of the entire endoderm and anterior mesoderm and suggest that this is mediated by its ability to enhance Nodal. As Arkadia facilitates Nodal signaling broadly, before and during gastrulation, it is likely to be a regular partner factor of the Nodal signal transduction pathway.

### Arkadia Acts Downstream of the Receptors and Destabilizes the Phosphorylated Forms of Smad2/3

**Figure 2:**
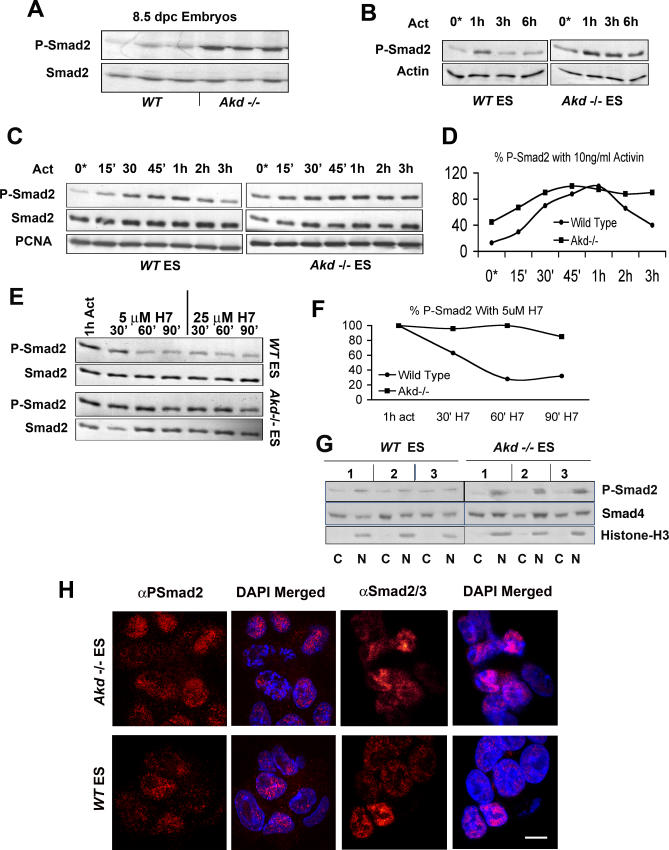
In the Absence of Arkadia, P-Smad2 Is More Stable and Correctly Localized in the Nucleus (A) Western blot of 8.5 dpc wild-type and *Akd*−/− embryo extracts. Each lane contains total protein extracts from three embryos. Note that P-Smad2 levels are at least 2-fold higher in mutants than in wild-type. (B and C) Western blot of extracts from *Akd−/−* and wild-type ES cells cultured with Activin for different intervals (in minutes (') and hours (h). Note that P-Smad2 is more stable in mutant than in wild-type cells. Loading control: PCNA, proliferation cell nuclear antigen. (D) Densitometry analysis of the bands on the blots in (C) showing the trend in P-Smad2 levels normalized against Smad2 where *Akd−/−*ES cells treated with Activin for 45 min is taken as 100%. (E) Western blot analysis showing P-Smad2 and Smad2 levels in ES cells stimulated with Activin for 1 h (1 h Act) and subsequently treated for different time points with H7 inhibitor as indicated. (F) Densitometry analysis of the blots in (E) showing the trend in P-Smad2 levels normalized against Smad2 where *Akd−/−* and wild-type ES cells treated with Activin for 1 h are represented as 100%. (G) Western blot showing P-Smad2 and Smad4 protein distribution in cytoplasmic and nuclear fractions of three different wild-type and *Akd−/−*ES cell lines as indicated. 30 μg of each protein sample was loaded for analysis. Histone H3, a nuclear protein, was used as a control for fractionation. C, cytoplasmic; N, nuclear. (H) Immunofluorescence with anti-P-Smad2 and anti-Smad2/3 (red) antibody on *Akd −/−* and wild-type ES cells treated with Activin A for 1.5 h prior to fixation, indicating no difference in the localization of P-Smad2 in the absence of Arkadia. Note the nuclear accumulation of P-Smad2, Smad2/3, and Smad4 in *Akd*−/− cells. White bar, 12 μm. WT, wild-type.

To find at what position within the Nodal signaling cascade Arkadia functions, we compared the level of the receptor-activated (phosphorylated) signaling effector P-Smad2 in embryos and embryonic stem (ES) cells by Western blotting (Figure 2). We examined 20 wild-type and 20 *Arkadia−/−* embryos at 8.5 days post-coitum (dpc) (Figure 2A and unpublished data) and three wild-type and three *Arkadia−/−* blastocyst-derived ES cell lines (Figure 2G). We found that while the total levels of Smad2 protein remain the same, P-Smad2 was always at least two times higher in all *Arkadia−/−* embryos and ES cells compared to the wild-type samples. Similarly, the other Nodal signaling effector P-Smad3 was found to be elevated in *Arkadia−/−*ES cell lines (Figure S1). Therefore, in embryonic cells, P-Smad2/3 are more abundant in the absence of Arkadia than in its presence. As the phosphorylation of Smad2/3 depends on the kinase activity of the ligand-activated receptors, the data suggest that in the absence of Arkadia the receptors are more active or that P-Smad2/3 are more stable after their phosphorylation.

We examined the activity of the receptors by stimulating *Arkadia−/−* and wild-type ES cells with Activin A ligand (Activin) and comparing Smad2 phosphorylation over time (Figure 2B–2D). Although *Arkadia−/−*cells start with higher basal levels of P-Smad2 compared to wild-type, Smad2 phosphorylation peaks 1 h after Activin addition in all ES cell lines (Figure 2B–2D), indicating normal receptor kinase activity in the presence or absence of Arkadia. Interestingly, after peak stimulation in wild-type ES cells, P-Smad2 decreases to basal levels within 2 h (Figure 2B–2D), but in *Arkadia−/−* ES cells P-Smad2 is maintained at peak levels (>90%) for at least 6 more h (Figure 2B). The data suggest that after receptor phosphorylation, Smad2 is more stable or maintains the phosphorylation longer in the absence of Arkadia. The total Smad2 levels do not change during the course of the experiment, indicating that the increased stability is associated with only the phosphorylated fraction (Figure 2C and 2D). To examine the possibility that other factors, such as a receptor inhibitor, is induced specifically in wild-type cells causing reduction of Smad2 phosphorulation, we repeated the above experiment in the presence of a protein synthesis inhibitor (cycloheximide). The decay of P-Smad2 was found, as before, to be slower in *Arkadia−/−* cells (Figure S2), suggesting that differences in protein stability rather than synthesis account for the increase in P-Smad2 levels.

To exclude the possibility that the receptors generate the differences in P-Smad2 levels, we blocked all the TGF-β receptors with the serine/threonine kinase inhibitor, H7 (Figure 2E and 2F) or used SB431542 selective inhibitor of Alk receptors (SB), which blocks the receptors that specifically phosphorylate Smad2/3 (Figure S1A). We found that in wild-type cells treated with two different concentrations (5 or 25 μM of H7), P-Smad2 levels declined 40% and 60%, respectively, within 30 min and they diminished to 30% after 90 min. In *Arkadia−/−* ES cells, however, even after 90 min, with the highest amount of inhibitor, P-Smad2 levels were not significantly changed (Figure 2F). The data indicate that the receptors are not responsible for generating the increase of P-Smad2 in *Arkadia−/−* cells and suggest that this is caused by P-Smad2 stabilization. The total Smad2 protein levels do not change during the course of the experiment (Figure 2E), but as the fraction of P-Smad2 is most likely small, differences within this fraction may not be visible when the total levels are examined. All of the above experiments were reproducible in three different *Arkadia−/−* ES cell lines (unpublished data) and the same results were obtained for the other Nodal/Activin effector, P-Smad3 (Figure S1B). Collectively, the data suggest that Arkadia acts downstream of the receptors and destabilizes the phosphorylated forms of Smad2/3.

### Smad4/P-Smad2/3 Are Nuclear in the Absence of Arkadia

P-Smad2/3 complex with Smad4 and translocate to the nucleus where they activate target genes and are subjected to different mechanisms of turnover and signaling termination such as ubiquitination/proteasome-mediated degradation [38] or de-phosphorylation [31] and nuclear export [50,51]. Cytoplasmic retention of P-Smad2/3 can result in both inability to activate target genes and increased stability. As in the absence of Arkadia, P-Smad2/3 are more stable and less transcriptionally efficient; it is possible that they are cytoplasmic and Arkadia regulates their nuclear localization. We therefore examined in three different wild-type and three *Arkadia−/−* ES cell lines the localization of P-Smads using Western blots of nuclear and cytoplasmic fractions (Figure 2G) or by immunofluorescence (Figure 2H) with antibodies against P-Smad2 or Smad2/3. We did not find evidence that P-Smads are cytoplasmic in the absence of Arkadia. On the contrary, the Western blots revealed that P-Smad2 accumulates in the nucleus of *Arkadia−/−*cells. Furthermore, as P-Smad2/3 complex with Smad4 to translocate to the nucleus [15], an increase of Smad4 in the nucleus of *Arkadia−/−* ES cells was observed (Figure 2G). Therefore, we conclude that Arkadia destabilizes P-Smad2/3 without affecting Smad4 complex formation and nuclear localization. Furthermore, as Arkadia is nuclear, the data suggest that Arkadia regulates P-Smad2/3 stability in the nucleus.

### Arkadia Interacts Directly with P-Smad2/3

**Figure 3:**
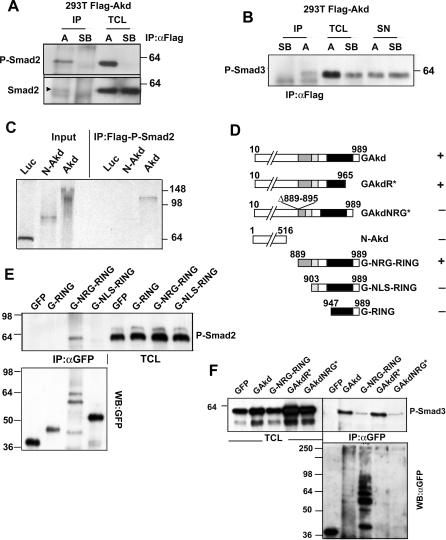
Arkadia Interacts Directly with P-Smad2/3 and This Requires the NRG Domain (A and B) IP with anti-Flag antibody from 293T cells stably expressing Flag-tagged Arkadia (Flag-Akd). Cells were cultured with Activin A (A) or SB and Western blotted with anti-P-Smad2 (A) or anti-P-Smad3 antibody (B). Note that Flag-Akd coIPs endogenous P-Smad2 (A) and P-Smad3 (B) in Activin and not in the SB-treated sample. Western blotting of IPs with anti-Smad2 antibody reveals this interaction to be specific to the phosphorylated form of Smad2 (A). The band in the total tissue cell lysates (TCL) and supernatants (SN) in SB-treated samples may correspond to P-Smad1 that does not coIP with Flag-Arkadia (B). Compare the intensity of the band in the TCL and SN to see the depletion of P-Smad3 after IP. (C) Autoradiograph showing in vitro transcribed/translated ^35^S-labeled full-length Arkadia (Akd), N-terminal portion (N-Akd), and luciferase (Luc) protein input. Flag-P-Smad2 (Figure S3B) attached to agarose beads was able to pull down specifically Akd and not N-Akd or Luc. (D) Map of Arkadia deletion constructs fused in frame with GFP on the N-terminus except for N-Akd. The numbers correspond to amino acid positions. NRG domain, dark gray; NLS, light gray; RING, black (see the amino acid sequence in Figure S3C). GAkdNRG* contains a partial internal deletion of the NRG domain (aa Δ 889–895). (E and F) IP with anti-GFP antibody from 293T cells transfected with GFP or GFP-tagged Arkadia constructs as indicated, along with Alk4* and either Flag-Smad2 (E) or myc-Smad3 (F) and Western blotted (WB) with antibodies as indicated. P-Smad2 protein is shown to coIP with the G-NRG-RING and not with other shorter forms missing the NRG domain. P-Smad3 protein is shown to coIP with full-length Arkadia (GAkd) and a RING domain deletion (GAkdR*). Note the presence of ladder and smear in GFP-tagged Arkadias including the G-NRG-RING, indicating that these proteins are unstable and heavily modified (including poly-ubiquitin chains; unpublished data).

Arkadia is an E3 ubiquitin ligase and could be destabilizing P-Smad2/3 directly by poly-ubiquitinating them, leading to their proteasome-dependent degradation. To test this hypothesis, we examined whether Arkadia interacts specifically with phosphorylated Smads. We used HEK293T (293T) cells stably expressing moderate levels of full-length Arkadia, tagged either with Flag on the N-terminus and Myc on the C-terminus, or with green fluorescent protein (GFP) fused to the N-terminus (GAkd). We performed immunoprecipitation (IP) with anti-Flag (Figure 3A and 3B), -Myc, or -GFP (unpublished data) antibodies and Western blotted with anti-P-Smad2, -Smad2 (Figure 3A), or -P-Smad3 (Figure 3B). The results show that with Activin stimulation, Arkadia coIPs with the phosphorylated endogenous Smad2 (Figure 3A) and Smad3 (Figure 3B). However, when the cells are treated with the SB receptor inhibitor, which eliminates Smad2/3 phosphorylation, Arkadia does not coIP unphosphorylated Smad2/3 (Figure 3A and unpublished data). Furthermore, we examined the interaction of Arkadia with other phosphorylated Smads (Smad1/5/8; Figure S3A) or with Smad4 (unpublished data) and found no evidence of interaction. We therefore conclude that Arkadia interacts specifically with the phosphorylated forms of Smad2/3.

To test how direct this interaction is, we performed the IP in vitro (Figure 3C) using in vitro transcribed/translated (recombinant) Arkadia protein labeled with S^35^ and phosphorylated Flag-tagged Smad2 isolated by IP (Figure S3B) from 293T cells stimulated with constitutive active Alk4 (Alk4*). The data show that phosphorylated flag-Smad2 protein can IP recombinant full-length Arkadia but not the N-terminal portion (1–510 amino acids [aa]), or the luciferase control (Figure 3C). The data suggest that Arkadia interacts directly with P-Smad2/3 via a C-terminal domain.

Arkadia is a 989-aa protein and its C-terminal half (516–989 aa) contains a highly conserved domain of 100 aa (889–989 aa), which includes the RING-ubiquitin ligase activity-domain at the C-terminal region (947–965 aa), a nuclear localization signal (NLS) (903–909 aa), and a conserved domain (889–903 aa), termed here NRG, of unknown function (Figure S3C). To understand the interaction of Arkadia with P-Smad2/3, we mapped further the responsible domain. We used transient transfections of 293T cells to test the ability of various deletions of Arkadia (all GFP-tagged; Figure 3D) to IP P-Smad2/3. The data show that the last 100 aa of Arkadia containing the NRG, the NLS, and the RING (G-NRG-RING, 889–989 aa; Figure 3D) are sufficient for the interaction (Figure 3D). Deletion of the C-terminal end of Arkadia (GAkdR*) that eliminates one of the Zinc-binding fingers of the RING domain (965–989 aa) does not affect the interaction with P-Smad2/3 (Figure 3F). However, deletion of the NRG completely abrogates the interaction with P-Smad2 (Figure 3E). To confirm that the NRG domain is necessary for the interaction with P-Smad2/3 within the context of the full-length Arkadia protein, we generated an internal partial deletion of only the first eight residues of the NRG (GAkdNRG*) and showed that it diminishes the interaction with P-Smad2/3 (Figure 3F). As judged by fluorescence from the GFP tag, all of the above mutant Arkadia proteins are localized in the nucleus and are expressed at comparable levels to full-length Arkadia (Figure S4), indicating that loss of the interaction of the various Arkadia deletion constructs is not due to instability or differential localization. Collectively, the above data indicate that Arkadia interacts directly with P-Smad2/3 via its 100-aa C-terminal portion and that within this domain, a 14-aa NRG motif is essential for this interaction. As the 293T cells that we used for the IPs do not express *FoxH1* (unpublished data), one of the major P-Smad2/3 transcription partners in early embryogenesis, we conclude that the interaction of Arkadia with P-Smad2/3 may not depend on a particular partner.

### P-Smad2/3 Are Substrates of Arkadia Ubiquitination

**Figure 4:**
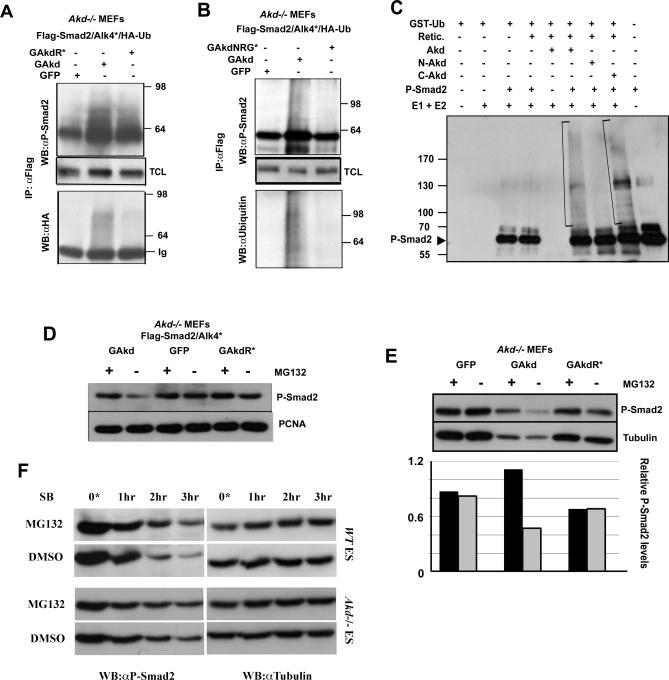
Arkadia Ubiquitinates P-Smad2 and Mediates Its Degradation via the Proteasome (A and B) IP with anti-Flag antibody from *Akd*−/− MEFs transiently transfected with various plasmids as indicated, and Western blotted (WB) with anti-P-Smad2, anti-HA, or anti-ubiquitin antibodies. Note the presence of higher molecular weight P-Smad2 corresponding to protein modifications, including ubiquitin chains as shown by the presence of HA-ubiquitin tags (A) or ubiquitin (B). These are present only in cells transfected with full-length Arkadia and not with GFP (A and B) or mutant forms GAkdR* (A) and GAkdNRG* (B), which disrupt the ubiquitin ligase activity and the interaction with P-Smad2, respectively. (C) In vitro poly-ubiquitinated P-Smad2 (bracket) was detected by Western blotting with anti-P-Smad2 antibody. Note the presence of high molecular weight species only in the reactions containing in vitro transcribed/translated full-length (Akd) or the C-Akd (aa 510–989) Arkadia proteins (see WB with anti-ubiquitin in Figure S5A). Retic., Reticulocyte extract; E1 + E2, ubiquitination enzymes; GST-Ub, GST-tagged ubiquitin. (D and E) Nuclear extracts from *Akd −/−* MEFs transfected with either GAkd, GFP, or GAkdR* plasmids alone (E) or with Flag-Smad2 and Alk4* (D) and treated with either MG132 (+ ; 30 μM) or DMSO (−) for 4 h prior to lysis, and Western blotted with anti-P-Smad2 and either anti-PCNA (D) or anti-Tubulin (E) antibodies for loading controls. P-Smad2 protein levels decrease specifically in cells expressing Arkadia, and this does not occur when the proteasome is inhibited or when the RING domain is mutated (GAkdR*), indicating that the degradation is mediated via the proteasome and the ubiquitin ligase activity of Arkadia. (E) Graphical representation of relative endogenous P-Smad2 levels normalized against the housekeeping gene Tubulin shows approximately a 2.5-fold reduction of P-Smad2 in Arkadia-expressing MEFs in the presence of DMSO when compared to MG132. GFP- and GAkdR*-expressing MEF samples do not show any differences. (F) Western blots (WB) with anti-P-Smad2 and anti-Tubulin antibodies showing the rate of P-Smad2 degradation in wild-type and *Akd−/−* ES cells in the presence of MG132 or DMSO control. After initial stimulation with Activin (0*), P-Smad2 is rapidly degraded in wild-type ES cells but not in *Akd−/−*. This degradation in wild-type cells is reduced in the presence of MG132 compared to the DMSO control, indicating that it is mediated via the proteasome. SB, SB431542; WT, wild-type.

As P-Smad2/3 interact with Arkadia, it is possible that they are substrates of Arkadia ubiquitination. To address this, we examined the ubiquitination status of P-Smad2 in the presence or absence of Arkadia expression. We used *Arkadia−/−* mouse embryonic fibroblasts lines (MEFs) to exclude any endogenous Arkadia activity and introduced Flag-Smad2 and Alk4* to obtain phosphorylated Flag-Smad2, in the presence of full-length or mutant forms of Arkadia. Western blot analysis of the IPs with anti-P-Smad2 antibodies showed the existence of higher molecular weight forms of P-Smad2 associated specifically with the presence of full-length Arkadia (Figures 4A and 4B and S4) suggesting poly-ubiquitination. Probing with ubiquitin antibodies confirmed that these modifications contain ubiquitin chains (Figures 4A and 4B and S4). Furthermore, these blots show that P-Smad2 is not ubiquitinated in the presence of mutant Arkadia proteins lacking either ubiquitin ligase activity (GAkdR*) or the P-Smad2 interaction domain (GAkdNRG*; Figure 4A and 4B). Therefore, Arkadia ubiquitinates P-Smad2 in vivo, and this depends on both its ubiquitin ligase activity and the P-Smad2/3 interaction domain, suggesting that Arkadia ubiquitinates them directly.

To verify that P-Smad2/3 ubiquitination is directly dependent on Arkadia, we performed the assay in vitro by adding all the components of ubiquitination separately along with recombinant full-length Arkadia, the C-terminal part of Arkadia containing the RING and the NRG, or N-terminal Arkadia. In this reaction we added the phosphorylated Flag-Smad2 obtained by IP as shown before (Figure S3B), and examined by Western blot, with anti-P-Smad2 antibody, whether or not it becomes modified by the ubiquitination reaction. We found that the full-length and C-terminal Arkadia are capable of poly-ubiquitinating in vitro Flag-P-Smad2, only when all the ubiquitination components were present, while the N-terminal Arkadia does not (Figures 4C and S5B). In addition, the Flag-P-Smad2 substrate does not become ubiquitinated without the addition of recombinant Arkadia, indicating that the IP is not contaminated with ubiquitin ligases from the cells. Collectively, the data show that P-Smad2/3 are ubiquitinated directly by Arkadia in vivo and in vitro, and therefore, they are qualified substrates of Arkadia ubiquitination.

### Arkadia Mediates Proteasome-Dependent Degradation of P-Smad2

Poly-ubiquitination of proteins usually leads to degradation via the proteasome [34,35]. Consistent with this, our data show that P-Smad2/3 are unstable in the presence of Arkadia (Figure 2), but it was unknown whether this instability is mediated by the proteasome. To address this, we transfected *Arkadia−/−* MEFs with full-length Arkadia or Arkadia lacking ubiquitin ligase activity under ligand stimulation and examined with an anti-P-Smad2 antibody the stability of transfected P-Flag-Smad2 (Figure 4D) or endogenous P-Smad2 (Figure 4E) in the presence or absence of MG132 proteasome inhibitor. We found that P-Smad2 levels are reduced specifically in the presence of full-length Arkadia and that MG132 can inhibit this. Therefore, Arkadia, via its ubiquitin ligase activity, is sufficient to induce P-Smad2 proteasome-dependent degradation.

In the above experiments, the degradation of P-Smad2 was achieved by the transfection of exogenous Arkadia in −/− MEFs. To examine whether endogenous Arkadia is necessary for proteasome-dependent degradation of P-Smad2, we compared its decay in *Arkadia−/−* and wild-type ES cells in the presence or absence of MG132. For this we first stimulated the ES cells with Activin (1 h), then added SB inhibitor to prevent further phosphorylation of Smad2 and examined its decay at different time points (Figure 4F). We found that MG132 protects P-Smad2 in wild-type ES cells but has very little effect in *Arkadia−/−* (Figure 4F). Therefore, Arkadia, by direct poly-ubiquitination, most likely mediates degradation of P-Smad2 by the proteasome.

### Arkadia Is Necessary and Sufficient to Enhance P-Smad2/3 Transcriptional Activity

**Figure 5:**
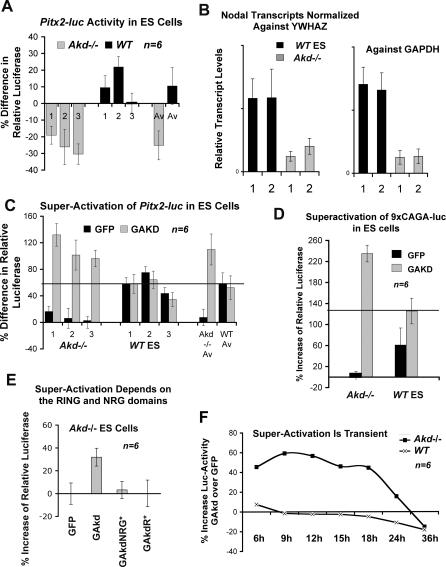
Arkadia Is Required for Efficient P-Smad2/3 Target Gene Transcription and Super-Activates Signaling in *Akd−/−* ES Cells (A) Relative luciferase activity from three different wild-type and three different *Akd−/−* ES cell lines transfected with the *Pitx2-luc* reporter. All values are expressed as a percentage relative to wild-type cell line 3 (3) represented as 0%. *Akd−/−* ES cell lines have lower activity compared to wild-type, indicative of reduced target gene expression. Average differences (Av) between wild-type and *Akd−/−* ES cells are shown. (B) *Nodal* real-time PCR performed on two different wild-type and *Akd−/−* ES cell lines (1 and 2). The abundance of endogenous *Nodal* transcripts was quantified and normalized against two different housekeeping control genes, *YWHAZ* and *GAPDH,* in quadruplicate reactions (*n* = 4 for each primer pair). Both *Akd−/−* ES cell lines show reduced levels of endogenous *Nodal,* a known target gene of P-Smad2/FoxHI compared to wild-type. (C) Percentage change in Nodal/TGF-β signaling as quantified by *Pitx2-luc* assays upon transient transfection of GFP or GAkd plasmids in three wild-type and three *Akd*−/− ES cell lines. GAkd expression doubles *Pitx2-luc* reporter activity in *Akd−/−* ES cells and the enhancement is 50% higher than the average level (Av) of wild-type ES cells (horizontal line). The values are normalized against GFP-transfected *Akd*−/− cell line 3, which has the lowest signaling. (D) Percent (%) increase in luciferase activity generated by the transiently transfected Smad3 specific *9xCAGA*-*luc* reporter in the presence of GFP- or GAkd-expressing plasmids in *Akd*−/− and wild-type ES cells showing that Arkadia super-activates Smad3-dependent transcription. The enhancement is 100% higher than that in wild-type ES cells (horizontal line). The luciferase values are expressed relative to that of *Akd−/−* cells transfected with GFP taken as 0%. (E) Super-activation is abolished when mutant Arkadias, GAkdR*, and GAkdNRG* are transfected, indicating that the enhancement requires Arkadia ubiquitin ligase activity and an interaction with P-Smad2/3. (F) Time course of super-activation shows that it is an early but transient phenomenon as quantified by the *Pitx2*-*luc* reporter. Relative luciferase activity of *Akd−/−* and wild-type ES cells are expressed as percent increase over the GFP control and compared at time points (h, hours) taken after transfection of GAkd or GFP. WT, wild-type.

We showed above that loss of Arkadia leads to the stabilization and nuclear accumulation of P-Smad2/3. But do these higher levels correspond to an increase in target gene transcription? Analysis of *Arkadia−/−* and compound *Arkadia−/−*, *Nodal+/−* embryos showed that Nodal signaling is defective (Figure 1 and [39,40]) suggesting that Smad2/3 target gene transcription is compromised. We examined the transcriptional activity of P-Smad2/3 in three *Arkadia−/−* and three wild-type ES cell lines. To estimate the relative levels of P-Smad2/3 transcriptional activity, we used two different target gene luciferase reporters, *0.9-P1,* (hereafter termed *Pitx2-luc*) regulated by P-Smad2/3 and its partner factor FoxH1, and *9xCAGA*–*luc,* a Smad3 specific reporter [52,53]. Although the *Arkadia−/−* ES cell lines always have a higher amount of P-Smad2 protein compared to wild-type, they have on average 30% (Figure 5A) lower luciferase from the wild-type cell line with the lowest activity (WT 3 designated as reference = zero). Stimulation with Activin did not change significantly the luciferase reporter expression (unpublished data); indicating that under standard culture conditions ES cells exhibit ligand-saturated signaling (autocrine signaling). In addition, real-time PCR showed that the expression of the endogenous *Nodal* gene, which like the *Pitx2*-*luc* luciferase reporter is regulated by FoxH1/Smad2/3 binding sites (known as ASE) [12], is reduced by about 70% in *Arkadia−/−* ES cells (Figure 5B). Together, these observations suggest that Arkadia is necessary for efficient target gene expression and suggest that in its absence, the stable and high levels of P-Smad2/3 are hypoactive or prevented from activating their target genes. Therefore, although Arkadia degrades P-Smad2/3, it is necessary for efficient P-Smad2/3 transcriptional activity.

To address whether Arkadia is sufficient to activate P-Smad2/3, we performed gain-of-function experiments in ES cells. We introduced full-length Arkadia (GFP-tagged; GAkd) in three −/− ES cell lines and showed that it enhances the expression of the luciferase reporters on average 100% (Figure 5C) and 230% (Figure 5D) above the level of a GFP-expressing plasmid. Interestingly, in wild-type ES cells, Arkadia does not significantly change the reporter activity (Figure 5C and 5D) even after Activin stimulation (unpublished data). This indicates that endogenous Arkadia is adequate to activate all P-Smad2/3 generated by the receptors. Together, these results suggest that Arkadia is necessary and sufficient to enhance P-Smad2/3 target gene transcription.

### Arkadia Enhances and Degrades P-Smad2/3 via the Same Domains

According to the above data, Arkadia has two functions: to destabilize P-Smad2/3 and enhance their activity. As it is possible that different domains mediate the two opposing functions, we used the −/− ES cell functional assay to identify domains that are essential for Arkadia to enhance reporter activity. We found that Arkadia constructs with ubiquitin ligase domain mutations (GAkdR* and GAkdR2*) or Arkadia without the P-Smad2/3 interaction domain (NRG deletion, GAkdNRG) fail to enhance (Figure 5E); suggesting that like the degradation of P-Smad2/3, activation also requires a direct interaction with Arkadia and its ubiquitin ligase activity. Examination of several N-terminal deletions (Figure 3D) showed that the C-terminal half (516–989 aa) of Arkadia is the minimum region sufficient to enhance the reporter efficiently (unpublished data). Furthermore, loss of the P-Smad2/3 activation, but not the degradation properties of Arkadia, is expected to convert it to a repressor of signaling. However, none of the deletions and mutations of Arkadia separated the two functions. Together, the above data suggest that Arkadia activates and degrades P-Smad2/3 via the same domains and that the two functions are most likely coupled.

### Enhancement of P-Smad2/3 by Arkadia Occurs at the Expense of Their Abundance

**Figure 6:**
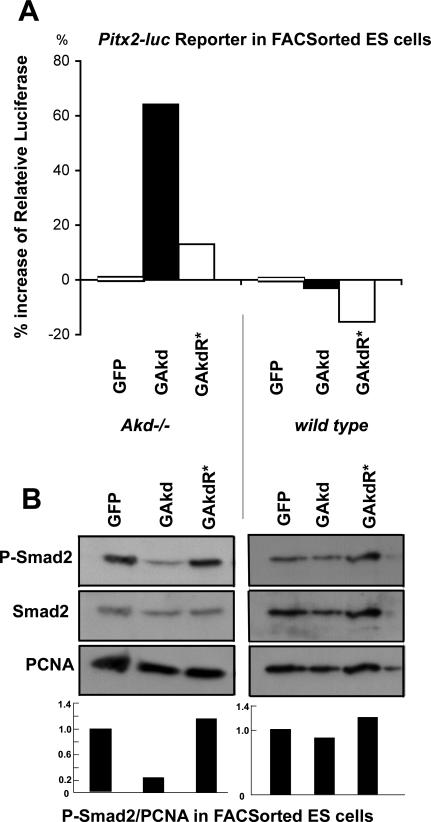
Activation of P-Smad2 Occurs at the Expense of Its Abundance Wild-type and *Akd*−/− ES cells transiently transfected with the *Ptx2*-*luc* reporter along with GFP, GAkd, or GAkdR* plasmids and isolated by FACS for GFP expression. Half of the cells were analyzed for luciferase activity (A) and the other half were Western blotted with anti-P-Smad2, -Smad2, and -PCNA (loading control) antibodies (B). Note that expression of full-length Arkadia results in super-activation and a reduction of around 80% of P-Smad2, only in *Akd*−/− ES cells, which have an accumulation of P-Smad2 (compare P-Smad2/Smad2 ratio). The phosphorylated Smad2 fraction is small as the ES cells are not stimulated (i.e., transfected with Alk4*), and therefore, the degradation of P-Smad2 is not reflected in the total level of Smad2. The graph represents P-Smad2 levels normalized against PCNA.

To test whether the activation of the hypoactive P-Smad2/3 by Arkadia occurs at the expense of their levels, we examined the relationship between levels of endogenous P-Smad2 and the degree of enhancement after expression of Arkadia in −/− ES cells. To visualize levels of endogenous P-Smad2, we isolated by fluorescence-activated cell sorting (FACS) pure populations of cells transfected with GAkd or enzymatically (ubiquitin ligase) inactive Arkadia (GAkdR*) or control GFP constructs. A portion of the cells was used for luciferase assays and the rest was used to examine endogenous P-Smad2 and total Smad2 levels in Western blots. As before, Arkadia enhances signaling only in −/− ES cells in a ubiquitin-ligase-dependent manner (Figure 6A); and this phenomenon was accompanied by an 80% reduction in the level of P-Smad2 (Figure 6B), confirming that activation of the hypoactive P-Smad2 is followed by its degradation.

We performed the same experiment in wild-type ES cells and found that overexpression of Arkadia does not change the levels of endogenous P-Smad2 or the reporter activity (Figure 6A and 6B). We conclude that in wild-type cells endogenous Arkadia must be in excess and sufficient to activate all available P-Smad2/3 in the nucleus. The fact that P-Smad2 is not eliminated and signaling is never repressed by the overexpression of Arkadia suggests that only a fraction of P-Smad2 interacts with and gets degraded by Arkadia, i.e., nuclear P-Smads and perhaps those engaged in transcription. The above data confirm the dual role and coexisting functions of Arkadia, suggesting that Arkadia enhances P-Smad2/3 activity at the expense of their levels.

### Super-Activation by Arkadia in −/− ES Cells Is a Transient Phenomenon

An interesting observation is that in −/− ES cells, Arkadia expression not only restores the transcriptional deficit but it enhances reporter activity on average 50% (line in Figure 5C) or 100% (Figure 5D) above the maximum level that can be achieved in wild-type ES cells. The simplest explanation for this phenomenon is that the extra activity most likely reflects the accumulated levels of the hypoactive P-Smad2/3 that is being simultaneously activated by the expression of Arkadia. As this enhancement exceeds the maximum that can be achieved in wild-type ES cells even under ligand stimulation conditions, we termed it super-activation. According to this hypothesis, Arkadia expression in −/− ES cells will eventually “consume” (activate and degrade) the accumulated P-Smad2/3, releasing their activity to produce a transient super-activation of target genes. Subsequently, target gene transcription will be reduced to basal levels similar to that of wild-type cells.

We tested this prediction by comparing the percentage of enhancement by GAkd over that of the GFP control at different time points after transfection in *Arkadia−/−* ES cells. The results indicate that maximum super-activation occurs as early as 9 h post-transfection and coincides with the appearance of GFP fluorescence, declines after 15–18 h, and disappears after 30 h (Figure 5F). GFP fluorescence remained high throughout the experiment and past 48 h (unpublished data) and does not account for the loss of super-activation at 30 h. The above data suggest that in −/− ES cells Arkadia releases the activity of the hypoactive and stable P-Smad2/3 pool causing target gene transcription above wild-type levels (super-activation), and as this is a transient phenomenon, it occurs at the expense of P-Smad2/3 abundance. Therefore, Arkadia functions by a mechanism that consumes P-Smad2/3 as it activates them.

### Arkadia Is Required for Nodal Target Gene Expression in the Embryo

**Figure 7:**
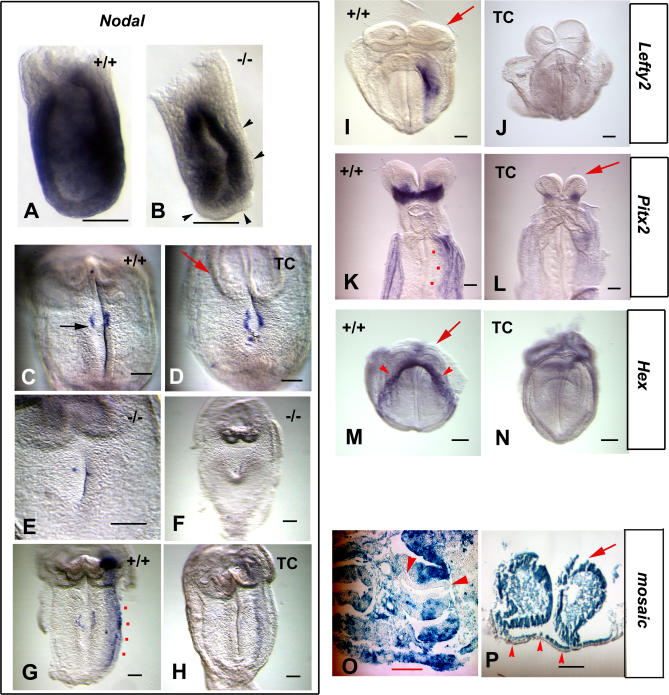
Arkadia Regulates Nodal Target Gene Expression and Phenocopies Smad2 in Development In situ hybridization with *Nodal* probe on 6.5 dpc (A and B) and 8.5 dpc (C–H) embryos; (C–H) embryos are shown as ventral views with anterior to the top and left toward the right; (A, C, and G) wild-type (+/+); (B, E, and F) *Arkadia*−/− (−/−); and (D and H) tetraploid chimeras (TC). Compare (A) and (B) to see reduced *Nodal* expression in the mutant embryo. The TC shows normal expression of *Nodal* around the node (D) and reduced expression in the left-LPM (H), while the −/− embryo has no node or *Nodal* expression (E and F). *Lefty2* probe on 8.5 dpc embryos (I and J) showing loss of expression in the left-LPM in the TC; *Pitx2* (K and L) on 9.5 dpc embryos showing reduction of expression in the TC; and *Hex* (M and N) on 8.5 dpc showing loss of expression in the foregut pocket of the TC. Heart-level sections from 9.5 dpc (O) and 8.5 dpc (P) mosaic embryos (chimeras) stained for β-galactosidase activity (blue). Note selective contribution of wild-type cells in the foregut. Black arrowheads point at the visceral endoderm; black arrow, points at the node; red dots indicate the left-LPM; red arrows point at the head folds; red arrowheads indicate the definitive endoderm at the level of the foregut. Bars, 0.1 mm.

All the above analysis shows that in ES cells Arkadia functions as a coactivator of P-Smad2/3 transcription. To address whether this also occurs in other embryonic cells and if this is the underlying cause of the *Arkadia−/−* phenotype in the embryo, we examined the expression of known Smad2 target genes in *Arkadia−/−* embryos. The FoxH1/P-Smad2 complex directly upregulates the *Nodal* gene and is responsible for its tissue-specific expression in the visceral endoderm (VE) at pre-gastrulation stages [10,12,54]. Whole mount in situ hybridization, as expected, revealed that in *Arkadia−/−* embryos (*n* = 10) *Nodal* expression is dramatically reduced in the epiblast and almost lost in the VE (Figure 7A and 7B).

Later in development, FoxH1/Smad2/3 regulates directly *Nodal* [12], *Pitx2,* and *Lefty2* [55,56] expression, in the left lateral plate mesoderm (LPM). However, this expression cannot be assessed in *Arkadia−/−* embryos because they lack a node and mesendoderm, which are essential for establishing left-right asymmetry. Using tetraploid chimeras (TC), we have previously shown that restoration of *Arkadia* expression in the extraembryonic lineages is sufficient to rescue the node and notochord formation in an embryo that consists entirely of *Arkadia−/−* cells [39]. We therefore generated TC embryos by injecting *Arkadia−/−* ES cells in tetraploid wild-type blastocysts. The rescue of node formation in the *Arkadia−/−* TC embryos is shown with the appearance of *Nodal* expression around the node (Figure 7D), which is absent in *Arkadia−/−* embryos (Figure 7E). We used these rescued *Arkadia−/−* embryos to examine target gene expression in the left LPM.

In the left LPM, expression of *Nodal, Lefty2,* and *Pitx2* is present in wild-type (Figure 7G, 7I, and 7K) and absent in *Arkadia−/−* embryos (Figure 7F), while in the TC embryos (*n* = 9), *Nodal* and *Pitx2* expression is severely reduced (Figure 7H and 7L) and *Lefty2* is undetectable (Figure 7J), indicating that Arkadia is required for Nodal target gene expression in the LPM. Furthermore, the expression of *Nodal* in the node of the TC embryos is not reduced (compare Figure 7C and 7D) and is consistent with previous findings that this expression does not depend on Smad2/3 [12,57]. This confirms that Arkadia is required specifically for the expression of Smad2/3 target genes in vivo. Therefore, in the absence of Arkadia, Nodal target gene expression is reduced broadly in many cells and tissues throughout early embryogenesis, before and during gastrulation, and can account for the developmental defects observed in the −/− embryos. In addition, the effect of Arkadia on *Nodal* gene expression can explain its non-cell autonomous functions in the *Arkadia*−/− TC embryos where wild-type VE (expressing Nodal) can rescue node formation, and those reported in the literature for *Xenopus* assays [39,40].

### Arkadia Phenocopies Smad2 and Is Required for Foregut and Prechordal Plate Formation

According to the biochemical and functional data in ES cells, Arkadia interacts with P-Smad2, and this interaction is essential for the full expression of target genes. It is therefore expected that *Arkadia−/−* embryos and ES cells will have similar defects and exhibit the same phenotypes with those of *Smad2*−/− cells. The phenotype of mice with conditional *FoxH1* or *Smad2* deletion exclusively in the epiblast shows that they cannot form ADE/future foregut or prechordal plate (the most anterior mesendoderm). Furthermore, injection of *FoxH1*−/− or *Smad2*−/− ES cells in wild-type blastocysts shows that the −/− cells cannot colonize tissues such as the gut and the prechordal plate, indicating a cell autonomous requirement for these factors in these chimeras (mosaic embryos) [58,59]. It is therefore expected that *Arkadia−/−* embryos and ES cells will have the same defect and exhibit the same phenotypes with those of *Smad2*−/− cells. Expression of *Hex,* an ADE/foregut marker [44] (Figure 7M and 7N) and *Shh,* a prechordal plate marker (Figure S6), show that although *Arkadia−/−* TC embryos develop node and mesendoderm, they exhibit a deficit in these tissues. Consistent with the role of the ADE and the prechordal plate in maintaining and patterning the head folds, we observed that the *Arkadia−/−* TC exhibit reduction of the head folds (Figure 7J, 7L, and 7N) that can also account for the reduction of *Pitx2* expression in the forebrain (Figure 7L).

The requirement for Arkadia expression within the cells that form the ADE/foregut and the prechordal plate was addressed in mosaic chimeras generated either by injection of wild-type ES cells into *Arkadia−/−* blastocysts (Figure 7O) or by *Arkadia−/−* ES cells into wild-type blastocysts (Figure 7P) [39]. In both types of chimeras, the embryo consists of a mixture of wild-type (unstained) and *Arkadia−/−* cells (β-galactosidase stained) [39]. We found that mosaic chimeras (*n* > 100) exhibit normal morphology, as long as wild-type cells colonize the foregut and the prechordal plate (Figure 7O and 7P). Therefore, like Smad2, Arkadia is required cell autonomously for ADE/foregut and prechordal plate formation. The above data show that Arkadia loss-of-function phenocopies that of Smad2 in embryonic cells, supporting a functional interaction between the two factors.

## Discussion

An important unanswered question is how the promoters of target genes are cleared and refreshed from transcription factors that are activated by signal transduction pathways to allow rapid intracellular responses to dynamic changes in the concentration of extracellular ligands. We show here that Arkadia terminates signaling at the level of transcription, by linking the ubiquitination/degradation of P-Smad2/3 to their transcriptional activity. Therefore, this mechanism enhances transcription but limits the time that the effector works by inducing its turnover. This represents the first example of coupled activation and degradation of signaling effectors, as well as a critical role for this mechanism in development. It is possible that very similar mechanisms operate in other pathways to establish dynamic regulation and efficient signaling, while their failure may be associated with disease.

### P-Smad2/3 Turnover

Experiments using somatic tissue culture cell lines showed that Arkadia might enhance TGF-β signaling by degrading the Smad6/7, which are known to inhibit Smad2/3 phosphorylation mainly by mediating the degradation of the receptors [41,42]. If this is the case, then embryos and ES cells should have reduced levels of P-Smad2/3 in the absence of Arkadia. Contrary to this prediction, all *Arkadia−/−* embryos and ES cells have at least 2-fold higher P-Smad2/3 levels compared to wild-type. Therefore, Arkadia must enhance signaling via a different mechanism during early development.

In vivo, in ES cells, MEFs, and embryos, loss of Arkadia causes P-Smad2/3 stabilization and accumulation in the nucleus and makes them resistant to proteasome degradation (Figure 4), suggesting that Arkadia directly or indirectly is associated with their stability and degradation. Destruction of P-Smad2 had been shown previously to occur in the nucleus [38], but a nuclear ubiquitin ligase that interacts specifically with the phosphorylated form of Smad2/3, as well as the role of proteasome-mediated turnover of P-Smads during development, remained unknown. We present here a number of experiments indicating that Arkadia, a nuclear E3 ubiquitin ligase, interacts with P-Smad2/3 and directly ubiquitinates them leading to their degradation by the proteasome. These include IP experiments showing that Arkadia interacts specifically with the phosphorylated forms of Smad2/3 via the conserved NRG domain (Figure 3A and 3B), and in vitro ubiquitination assays showing that Arkadia directly poly-ubiquitinates P-Smad2 (Figures 4A–4C and S5). Furthermore, introduction of Arkadia in −/− cells causes P-Smad2 poly-ubiquitination and decreases its abundance in a proteasome-dependent manner (Figure 4D–4E). However, as Arkadia is nuclear, it most likely degrades nuclear P-Smads.

### Signaling Termination at the End of the Cascade

One of the major questions is how effector activity is terminated after target genes have been activated and not before. This should involve a mechanism to distinguish between effectors actively engaged in transcription and those that are fresh and unused. A modification or a change of conformation of the effectors when they interact with the target-gene promoter complex may allow recognition by a critical component of the termination mechanism (referred to subsequently as the terminator). In this case, absence of the terminator should lead to a prolonged, persistent response to signaling, as the unhindered effectors will continue to transcribe. On the other hand, excess terminator could cause repression of signaling if it degrades and deactivates the effectors prematurely, i.e., as soon as they bind to the promoter. A more precise and efficient mechanism would involve degradation of the effectors after they activate at least one round of transcription. This requires a terminator directly involved in transcription by the effectors or an even more stringent mechanism, where initiation of transcription is linked with the destruction of the transcription factor: a “suicide” model. In this case, absence of the terminator will pause transcription, while its overexpression will not degrade/deactivate the effectors prematurely to repress target gene transcription. We show here that Arkadia fulfils the criteria of such a termination mechanism as it degrades P-Smad2/3 and enhances their target gene transcription via the same domains.

### P-Smad2/3 Activation

Does Arkadia directly activate P-Smad2/3? In the absence of Arkadia, P-Smad2/3 are stable and accumulate in the nucleus, but they are repressed or hypoactive as target gene transcription is reduced and in some cases lost (Figure 7). Introduction of Arkadia in −/− ES cells not only restores signaling but super-activates P-Smads, meaning that the expression of the reporter reaches levels higher than those that can be achieved in wild-type ES cells under maximum ligand stimulation conditions. The above findings suggest that the extra activity is not generated by receptors or ligands, but by the release of activity from the pools of the accumulated hypoactive P-Smad2/3. Consistent with this hypothesis, super-activation by Arkadia in −/− ES cells is a transient phenomenon, as it depends on the excess P-Smad2 that is used up. Therefore, Arkadia degrades and spends P-Smad2/3 to activate their transcriptional activity. These observations suggest that P-Smad2/3 is reminiscent of fuel, which in the absence of Arkadia is stable and can be stored, while in its presence it is “ignited” and “burns,” releasing activity. These observations suggest that phosphorylation of Smad2/3 by the receptors is not sufficient for their transcriptional activity and that they require an additional step mediated by the function of Arkadia as a coactivator in the nucleus.

We have shown previously that in *Xenopus* animal cap assay, injection of Arkadia RNA enhances the ability of Nodal but not that of Smad2 RNA to induce mesendoderm [40]. This appears inconsistent with our current data, however; as in the animal cap there is no ligand to phosphorylate Smad2, it remains a mystery how Smad2 RNA injection alone induces mesoderm and mesendoderm. Furthermore, the amount of Smad2 RNA required for this is very high and most likely does not represent physiological signaling conditions. More importantly, we show here that Arkadia recognizes P-Smad2 and not Smad2, and therefore it is not surprising that in the Smad2-injected animal caps Arkadia does not induce mesendoderm.

While the exact mechanism of how P-Smad2/3 are activated by Arkadia remains unknown, it is important to point out that Arkadia does not degrade P-Smads directly, it only modifies them by ubiquitination and is the proteasome that degrades them. Although the role of ubiquitin modification in mediating proteasome degradation is well established, it has become apparent that addition of ubiquitin onto proteins may also affect their properties. Recent studies have suggested a direct role of ubiquitin modifications and of the proteasome in transcriptional activation (reviewed in [60–63]). It is possible, however, that Arkadia interacts with, and degrades simultaneously, P-Smads and a closely linked repressor. Future studies will determine the mechanism of P-Smad2/3 activation and how this is coupled with immediate degradation. In conclusion, by linking P-Smad2/3 activation with degradation, Arkadia provides a way to achieve rapid resetting of P-Smad2/3 target gene transcription in development and allows efficient and dynamic responses to Nodal/TGF-β signaling events.

### Nodal Expression and the Induction of the Node/Organizer

We had shown previously that *Arkadia−/−* embryos do not develop anterior primitive streak and its derivative tissues: ADE/future foregut, mesendoderm (prechordal plate and notochord), and node [39]. However, the molecular mechanisms that underlie these abnormalities were not understood. Specifically, we had shown that the formation of the node is restored in chimeric embryos consisting of tetraploid wild-type extraembryonic lineages (such as VE) and exclusively *Arkadia−/−* embryonic tissues derived from −/− ES cells [39]. This indicates that the development of the node in the embryonic lineages requires Arkadia expression and function within a different lineage (extraembryonic). As Arkadia is nuclear and unlikely to be secreted, this effect was presumed to be mediated by a downstream-secreted factor. Here we show that Arkadia increases directly the transcription of P-Smad2 target genes, which include the *Nodal* gene itself, particularly in the VE and early epiblast when node precursors are induced (Figure 7). This suggests that in the *Arkadia−/−* TC, the wild-type VE provides sufficient Nodal to partially restore the overall level of Nodal in the *Arkadia−/−* epiblast, thus bringing it up to the threshold required for node formation (Figure 7D and unpublished data). Therefore, organizer/node induction requires high levels of Nodal, which can be achieved with a considerable contribution from the VE.

### Arkadia in Foregut Development

While the node may be rescued, to what extent expression of Arkadia in the extraembryonic lineages is sufficient to restore normal development in the *Arkadia−/−* embryo of the TC? We show here that these embryos cannot form the most anterior derivatives of the primitive streak, such as ADE/foregut and prechordal plate (Figure 7), and exhibit laterality defects, including delayed turning and heart looping ([39] and unpublished data). This is consistent with our findings that Arkadia is required for efficient P-Smad2/3 target gene transcription. Specifically, the reduction of *Nodal* expression in the left LPM along with its known target genes *(Lefty2* and *Pitx2)* can account for the left-right axis defects. The Nodal target genes that are responsible for foregut and prechordal plate development remain unknown. However, the development of these tissues must depend on the expression of Arkadia within these cells or their precursors. This was shown by the analysis of chimeric embryos consisting of a mixture of wild-type and *Arkadia−/−* cells, which revealed that the −/− cells could not contribute to these tissues (Figure 7O and 7P). Furthermore, as *Smad2*−/− cells [59] and *FoxH1*−/− cells [58] behave similarly in chimeras, our data provide in vivo evidence for the functional interaction between Arkadia and Smad2, and reveal that the three factors together regulate target genes essential for foregut endoderm and prechordal plate formation.

### How Is Maximum Nodal Signaling Achieved?

Our data show that Arkadia is essential for the development of tissues that depend on very high Nodal signaling such as ADE/foregut, and that its introduction in −/− ES cells can boost signaling above levels obtained by just high concentrations of ligand “super-activation.” This latter phenomenon is presumably generated by the simultaneous activation of the accumulated P-Smad2/3, which has been stabilized and reached higher than normal levels in the absence of Arkadia. It is possible that such a mechanism may be responsible for maximizing Nodal signaling in the embryo. However, it would require the transient absence of Arkadia in the precursors of the ADE/foregut. Although Arkadia RNA is present broadly in the embryo, protein analysis is needed to reveal whether super-activation occurs in the embryo.

In conclusion, we reveal a novel ubiquitin-mediated mechanism of TGF-β signaling regulation that depends on Arkadia, involves the activation of P-Smad2/3 signaling effectors downstream of receptor-phosphorylation, and couples their high activity with turnover and signaling termination. As the TGF-β/Nodal signaling pathway has been linked to cancers and genetic diseases, the identification of key molecular players such as Arkadia would be useful for the development of new drug targets and therapeutic intervention.

## Materials and Methods

### Expression analysis.

Chimeras were generated as described before [39]. Both adult mice and embryos were genotyped using allele-specific PCR amplification of genomic DNA. The oligonucleotide primer pairs TGAGGTAGGCATACCTAGAG and TGACTTAAGCCCTGCAATCC; TGAGGTAGGCATACCTAGAG and CTGAGTGATTGACTACCCGT; and TCTGGATTCATCGACTGTGG and CTGGATGTAGGCATGGTTGGTAG were used to give diagnostic amplification products of 313 bp for the wild-type *Arkadia* allele, 293 bp for the disrupted *Arkadia* allele, and 925 bp for the disrupted *Nodal* allele, respectively. Histology and in situ hybridizations and marker plasmids used are as previously described [39].

### RNA extraction and real-time PCR.

Total RNA was extracted from ES cells using Trizol (Invitrogen, http://www.invitrogen.com) followed by digestion with RQ1 RNase-Free DNase (Promega, http://www.promega.com) to remove DNA contamination. Synthesis of cDNA from total RNA was performed with SuperScript II Reverse Transcriptase (Invitrogen). Experiments were performed in quadruplicates using the DNA Engine Opticon Real-Time PCR Detection System (MJ Research, http://www.bio-rad.com) and SYBRGreen PCR Master Mix (Applied Biosystems, http://appliedbiosystems.com). The Nodal 375-bp amplicon was produced by the forward/reverse primer pair AAGACCAAGCCACTGAGCAT and GCCTTTGCACACAATTTCAA. Nodal expression was quantified by normalizing against endogenous controls GAPDH (primer pair TGCACCACCAACTGCTTAGC and GGCATGGACTGTGGTCATGAG) or YWHAZ (primer pair CGTTGTAGGAGCCCGTAGGTCAT and TCTGGTTGCGAAGCATTGGG) using the delta Ct method.

### DNA constructs.

Tagged Arkadia: Mus musculus ring finger 111 (Rnf111) constructs were generated by eliminating the first 9 aa of Arkadia and fusing in frame to GFP (pEGFP-cI; Clontech, http://www.clontech.com) or Flag-tag (synthetic oligonucleotide). A single Myc-tag (synthetic oligonucleotide) was fused to the carboxy-terminus of Arkadia. The various tagged Arkadia sequences and the GFP control gene were subcloned into SmaI/XhoI of the pTriEx2-hygro (Novagen, http://www.novagen.com) vector. The Arkadia RING domain mutations were constructed by either deleting the last 24 aa that included the second Zinc finger (GAkdR*) or by point mutation leading to amino acid substitutions (C2–A2) that disrupt both Zinc fingers (GAkdR2*). GAkdNRG* consists of an internal deletion of the first 7 aa of the NRG domain by PCR. The truncated Arkadia containing only the C-terminal region of Arkadia was constructed by fusing GFP in frame with the N-terminus of the truncated Arkadias. G-NRG-RING includes aa 889–989; G-NLS-RING, 903–989; GRING 947–989. *Pitx2-luc* and *123-luc* (gifts of H. Hamada, Osaka, Japan) and *9xCAGA-luc;* 6 Myc-Smad3; constitutively active forms of Alk4 and Alk6 (gifts of K. Miyazono, Tokyo, Japan); HA-Ub (gift of Y. Fujita, London, United Kingdom); and the Flag-Smad2 construct (gift of J. Smith, Cambridge, United Kingdom).

### Cell culture and luciferase assays.

The ES cell lines used in this study were derived directly from blastocysts as described previously [9]. Subsequently, they were maintained feeder-free in 20% FCS in DMEM and LIF (Invitrogen). Cells were transiently transfected with Lipofectamine 2000 (Invitrogen), Lipofectamine Plus (Invitrogen), or TransIT-293 (Mirus, http://www.mirusbio.com). *Arkadia* null primary mouse fibroblasts of mixed 129Sv/MF1 genetic backgrounds were isolated from embryos at 9.5 dpc and immortalized with *pBabe-puro-SV40-TA* (gift from Parmjit Jat, Ludwig Institute, London, United Kingdom), selected with 0.3 μg/ml puromycin and maintained in F12 (Invitrogen) medium with 20% FCS. Signaling stimulation was achieved using 50 μng/ml BMP2 or 10 μng/ml Activin A (Sigma, http://sigmaaldrich.com); signaling inhibition using serine/threonine kinase inhibitor H7 (Calbiochem, http://www.calbiochem.com) or SB431542 (Sigma and gift from GSK); and translation inhibition using 100 μg/ml cycloheximide (Sigma).

Cells were cotransfected with the Firefly reporter plasmids, a Renilla luciferase transfection control *pRL-SV40* (Promega), and various GFP-tagged *Arkadia* constructs in a 5:1:5 ratio. Dual luciferase assays were performed according to manufacturer's protocols (Promega) 18 h after transfection, unless stated otherwise. After normalizing the Firefly values to Renilla, the data was presented as relative luciferase values or as percent increase.

### Protein analysis.

For IP studies, cells in 10-cm culture dishes were treated for 5 h with 50 μM of the proteasome inhibitor MG-132 (Calbiochem) before lysis. The cells were lysed by adding 1 ml per 10-cm dish of RIPA (150 mM NaCl, 50 mM Tris [pH 8.0], 0.5% DOC, 0.1% SDS, and 1% NP-40) or NP-40 buffer (20 mM Tris [pH 7.5], 150 mM NaCl, 0.5% NP-40), which was specifically used for the IPs shown in Figure S5A. Both IP solutions contained 10 mM *N*-ethylmaleimide (cysteine protease inhibitor, Sigma), 100 μM MG-132 (Calbiochem), 100 μM Epoxomicin (Calbiochem), 100 μM *clasto*-Lactacystin β-Lactone (Calbiochem), and protease/phosphatase inhibitor cocktails (Sigma) at a final concentration of 1% each. After centrifugation at 100,000 *g* for 30 min, the supernatants were immunoprecipitated for 1 h with the appropriate antibody and 50 μl of protein agarose beads (Sigma). 2 μg of an anti-GFP mouse monoclonal antibody (clone 7.1 and 13.1; Roche, http://www.roche.com) or 2 μg of the anti-FLAG M2 antibody (Stratagene, http://www.stratagene.com) with 50 μl of Protein G Sepharose beads (Amersham Biosciences, http://www.gehealthcare.com) was used for the IP. Samples were eluted off the beads by boiling in 2× Laemelli buffer followed by standard SDS-ployacrylamide gel electrophoresis (SDS-PAGE) and Western blot analysis.

For nuclear and cytoplasmic fractionation, cells were lysed in hypotonic buffer (20 mM Hepes [pH7.5], 10 mM NaCl, 0.2 mM EDTA, 20% glycerol, 1.5 mM MgCl2, 0.1% Triton X-100) containing protease and phosphatase inhibitors as above. Nuclei were pelleted by centrifugation at 1,000 rpm for 10 min, and the cytoplasmic fraction obtained by retaining the supernatant. Nuclear extracts were obtained by rocking the nuclear pellet in five times the volume of hypertonic solution (hypotonic buffer + 500 mM NaCl) for 1 h at 4° C and subsequent centrifugation at 13,000 rpm for 5 min. Nuclear fraction was obtained in the supernatant, and 30 μg of each protein sample was loaded on each lane. When required to inhibit protein degradation, MG-132 (50 μM) was added to both the hypotonic and hypertonic buffers.

### Western blot analysis.

For Western blotting, cells and embryos were lysed with the RIPA buffer as for IPs. 30 μg of each protein sample was loaded on each lane for SDS-PAGE. The following antibodies were obtained and used according to the manufacturer's instructions: rabbit anti-P-Smad2 (Calbiochem), rabbit anti-P-Smad3 (Cell Signaling Technologies, http://www.cellsignal.com and also gift from E. Leof, Mayo Clinic, United States), rabbit anti-P-Smad1/5/8 (Cell Signaling Technologies), rabbit anti- Smad2 (Zymed Laboratories, http://www.invitrogen.com), rabbit anti-GFP (Abcam, http://www.abcam.com), rabbit anti- cyclin D2 (H-295; Santa Cruz Biotechnology, http://www.scbt.com), as well as mouse monoclonal antibodies against Smad4 (B-8; Santa Cruz); actin (Santa Cruz); PCNA (PC-10; Santa Cruz); tubulin (B-5-1-2; Sigma); FLAG (M2; Stratagene); histone H3 (Upstate, http://www.upstate.com); ubiquitin (Covance and Bethyl Laboratories, http://www.bethyl.com) and HA (Roche).

### In vitro interaction and ubiquitination assays.

[^35^S]-methionine-labeled or unlabeled recombinant proteins full-length Arkadia, N-Akd (1–516 aa), C-Akd (510–989), and luciferase were generated by the TNT Quick Coupled in vitro transcription/translation kit (Promega). Flag-P-Smad2 was obtained with IP from 293T cells. Beads bound to Flag-P-Smad2 protein were washed with RIPA buffer and incubated for 1 h by constant rotation at 4 °C with radiolabeled recombinant proteins in 1 ml of RIPA buffer. Protein-bead complexes were then washed four times with RIPA buffer (200 × bed volume) and re-suspended in 2 × Laemelli buffer. The presence of radiolabeled Arkadia protein in pull-downs was detected by SDS-PAGE and autoradiography. Assays were carried out in 20 μl of ubiquitination assay buffer (20 mM Tris-HCl [pH7.7], 100 mM KCl, 0.1 mM CaCl2, 1 mM MgCl2, 1 mM DTT) containing 15 μg of GST-ubiquitin (Boston Biochem, http://www.bostonbiochem.com), 1 μg of E1 (Boston Biochem), 1.5 μg of E2 (GSTUbcH5b and GST-UbcH5c; Boston Biochem), 0.6 μl of Energy Regeneration Solution (Boston Biochem), and Flag-P-Smad2 immunoprecipitated from 293T cells. In vitro translated Arkadia and Arkadia mutants (N-terminal corresponding to aa 1–516 and C-terminal truncation corresponding to aa 510–989) were generated using the TNT Quick Coupled in vitro transcription/translation kit (Promega). Reactions were incubated for 1 h under constant rotation at 37 °C. The reaction was terminated by the addition of 2 × Laemelli buffer, and the presence of poly-ubiquitinated substrates was detected by Western blotting.

### Immunofluorescence in ES cells.

Immunofluorescence was performed using the following primary antibodies: anti-P-Smad2 antibody (1:50; Calbiochem); anti-Smad2/3 (1:100; BD Biosciences, http://www.bdbiosciences.com); Alexa Fluor 568 anti-rabbit secondary (1:400; Molecular Probes, http://www.invitrogen.com); and mounting medium with DAPI (Vectashield, http://www.vectorlabs.com). *Arkadia−/−* and wild-type ES cells were grown on cover slips and rinsed in PBS for 5 min prior to fixation with 4% (w/v) paraformaldehyde in PBS for 10 min. Following fixation, cells were permeabilized with 0.5% Triton-X 100 in PBS for 2 min on ice and rinsed with PBS for 5 min and incubated in 10% FCS/PBS for 30 min to block nonspecific binding of antibodies. Subsequently, the cells were incubated with the appropriate primary antibody diluted in 10% FCS/PBS for 1 h at room temperature. A no primary control was included to verify antibody specificity. Cover slips were then washed several times with 10% FCS/PBS and incubated with secondary antibody for a further 1 h. This was followed by washes in PBS and cover slips mounted for fluorescence in medium containing DAPI (Vectashield). The cells were visualized on a Leica TCS SP2 (http://www.leica.com) confocal microscope at 100× magnification.

## Supporting Information

Figure S1 P-Smad2/3 Are More Stable in *Arkadia* Null ES Cells than in Wild-Type Western blots of wild-type and *Akd −/−* ES cells showing rate of decay of P-Smad2/3 at different time points during treatment with 10 μM SB inhibitor (A) or 20 μM H7 inhibitor (B). At point 0* the inhibitors were added after stimulation with Activin (A and B) and BMP2 (B) for 1 h. Note that the levels of P-Smad2 (A) and P-Smad3 (B) protein at 2 and 3 h in *Akd −/−* are higher than in wild-type ES cells. Western blotting with the anti-PCNA antibody show similar amounts of protein was loaded for each sample. The experiment is reproducible with different ES cells lines (unpublished data).
(757 KB TIF)

Figure S2 The Decay of P-Smad2 Is Slower in *Arkadia−/−* ES Cells in the Absence of Protein Synthesis Western blot of *Akd−/−* and wild-type ES cells cultured with Activin A and cycloheximide for different periods in hours (h). Note that P-Smad2 is more stable in mutant than in wild-type cells, while in both cell lines Cyclin D2 levels decline similarly and Tubulin remains stable throughout the treatment.
(1.5 MB TIF)

Figure S3 Arkadia Interacts Specifically with P-Smad2/3 and Not with P-Smad1/5/8 (A) IP with anti-GFP antibody from 293T cells transfected with ALK6* and all three myc-Smad1/5/8 plasmids, along with GFP or GFP-tagged Arkadia constructs as indicated, and Western blotted with anti-P-Smad1/5/8 or anti-GFP antibodies. Similar levels of P-Smad protein were present in the total cell lysates (TCL), but were absent in the IP lanes, indicating lack of interaction. Note the presence of high molecular weight proteins in the samples with the various GFP-tagged Arkadias (red arrowhead).
(B) Aliquot of the P-Smad2 protein used for in vitro pull-down (Figure 3C) and ubiquitination assays (Figure 4C). P-Smad2 isolated by IP with anti-Flag antibody from 293T cells cotransfected with Flag-Smad2 and ALK4* or in the presence of SB, and Western blotted with anti-P-Smad2 antibody. Note that phosphorylated Flag-Smad2 is present in the IP from cells with Alk4* but not with SB.
(C) Sequence of the C-terminal region of Arkadia (from aa 889 to terminal 989) containing the NRG domain (blue), a nuclear localization signal (NLS; green), and a RING domain (red). Critical residues (C3H2C3) comprising the RING domain structure are in red (cysteines) or blue (histidines).
(3.4 MB TIF)

Figure S4 Mutant Arkadia Proteins Localize in the Nucleus or in Both the Nucleus and Cytoplasm (A) 293T cells transiently transfected with plasmids expressing GFP-tagged N-terminal Arkadia deletions.
(B) 293T cells stably expressing GAkd, GAkdR*, or GAkdNRG*. Confocal microscopy was used to visualize GFP and DAPI nuclear stain.
(6.5 MB TIF)

Figure S5 Arkadia Directly Ubiquitinates P-Smad2 (A) IP with anti-Flag antibody from *Akd −/−* MEFs transiently transfected with various plasmids as indicated, and Western blotted (WB) with anti-P-Smad2, anti-Smad2, or anti-HA antibodies. Note the presence of higher molecular weight P-Smad2 corresponding to protein modifications including ubiquitin chains as shown by the presence of HA-ubiquitin tags. These are present only in cells transfected with full-length Arkadia and not with GFP or mutant forms GAkdR* and GAkdNRG*, which disrupt the ubiquitin ligase activity and the interaction with P-Smad2, respectively.
(B) Western blot with anti-P-Smad2 and -Ubiquitin antibodies of in vitro ubiquitination reactions containing various components as indicated. Arkadia was generated from reticulocyte extracts and Flag-P-Smad2 protein derived from 293T cells transiently transfected with Flag-Smad2 and Alk4* (Figure S3B). Note the increase of high molecular weight Flag-P-Smad2 forms only in the reaction containing Arkadia. The presence of high molecular weight ubiquitinated proteins in the reaction containing Arkadia without P-Smad2 indicates that Arkadia self-ubiquitinates, while the presence of more ubiquitinated forms in the reaction that contains both Arkadia and P-Smad2 indicates that both proteins are ubiquitinated.
(3.0 MB TIF)

Figure S6 *Arkadia* Null TCs Exhibit Prechordal Plate Deficit In situ hybridization with *Shh* riboprobe on 9.5 dpc (WT, A and C) and TC (B and D). (C and D) Section level of the embryos shown in (A and B) is indicated by the blue line. Note in the TC the head truncation and the reduction of *Shh* expression in the anterior region corresponding to the prechordal plate (B), the presence of notochord (NC), and aberrant gut tube endoderm (GE) in (D).
(5.0 MB TIF)

### Accession Numbers

The GenBank (http://www.ncbi.nlm.nih.gov/genbank) accession numbers for the entities from the discussed in this paper are *Arkadia* (NM_033604), *GAPDH* (NM_001001303), *Nodal* (NM_013611), and *YWHAZ* (NM_011740).

## Abbreviations

aa: amino acid
ADE: anterior definitive endoderm
Alk4*: constitutive active Alk4 receptor
AVE: anterior visceral endoderm
dpc: day post-coitum
GAkd: N-terminal GFP-tagged Arkadia
GAkdNRG*: N-terminal GFP-tagged Arkadia with the NRG domain deleted
GAkdR*: N-terminal GFP-tagged Arkadia with a mutation in the RING domain
GFP: green fluorescent protein
G-NRG-RING: N-terminal GFP-tagged Arkadia containing the NRG, NLS, and RING domains
IP: immunoprecipitation
LPM: lateral plate mesoderm
MEF: mouse embryonic fibroblast
NRG: the first conserved amino acids of the domain
P-Smad: phospho-Smad
SB: SB431542 Selective Inhibitor of Alk Receptor
TC: tetraploid chimera
TGF-β: transforming growth factor-β
VE: visceral endoderm
